# Isolated gallbladder hematoma after a blunt abdominal trauma: case report

**DOI:** 10.1186/2036-7902-7-S1-A27

**Published:** 2015-03-09

**Authors:** I-Ting Wang, Ming-Tse Tsai, Chun-Yen Huang, Kuang-Chau Tsai, Shih-Hao Wu, Wan-Ching Lien, Jen-Tang Sun

**Affiliations:** 1 Department of Emergency Medicine, Far Eastern Memorial Hospital, New Taipei City, Taiwan; 2 Department of Emergency Medicine, Chang-Gung Hospital Lin-Ko Branch, Taoyuen, Taiwan; 3 Department of Emergency Medicine, National Taiwan University Hospital, Taipei City, Taiwan

**Keywords:** Blunt Abdominal Trauma, Intramural Hematoma, Serum Glutamic Pyruvic Transaminase, Gallbladder Perforation, Solid Organ Injury

## Background

Sonography has played an important role on detection of intraabdominal injuries with a sensitivity of 86%, a specificity of 98%, and an accuracy of 97% [1]. Negative predictive value achieved about 98% [1]. However, gallbladder injury is rare with incidence around 2.1% [2, 3] in blunt abdominal trauma [2], and commonly associated with the solid organ injury or abdominal vascular injury [4]. Isolated gallbladder injury were very few[3] including traumatic cholecystitis, gallbladder tear, and gallbladder perforation ...etc. Ultrasound is very sensitive to gallbladder disease. We presented a rare case of isolated gallbladder hematoma after a blunt abdominal trauma which diagnosis by Focused Gallbladder ultrasound.

## Case report

**Figure 1:**
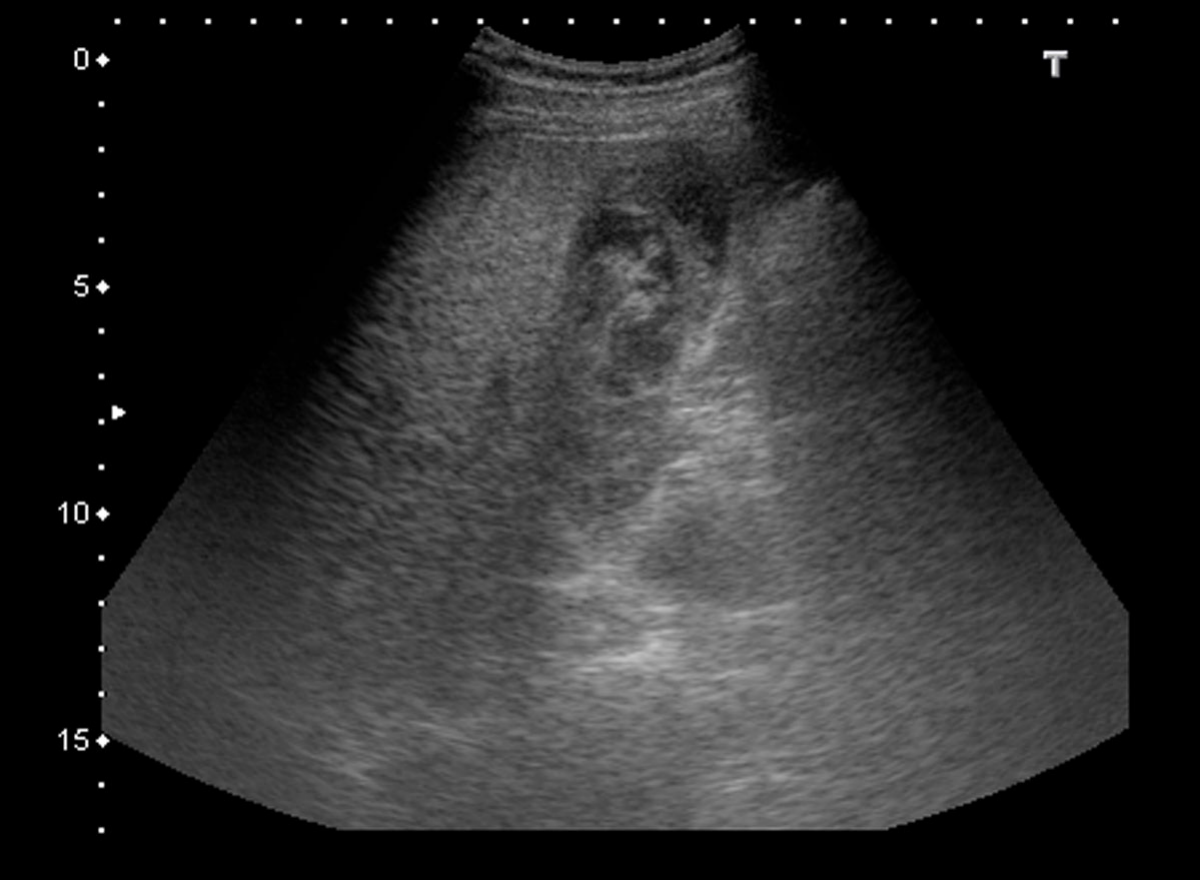
Figure 1

**Figure 2:**
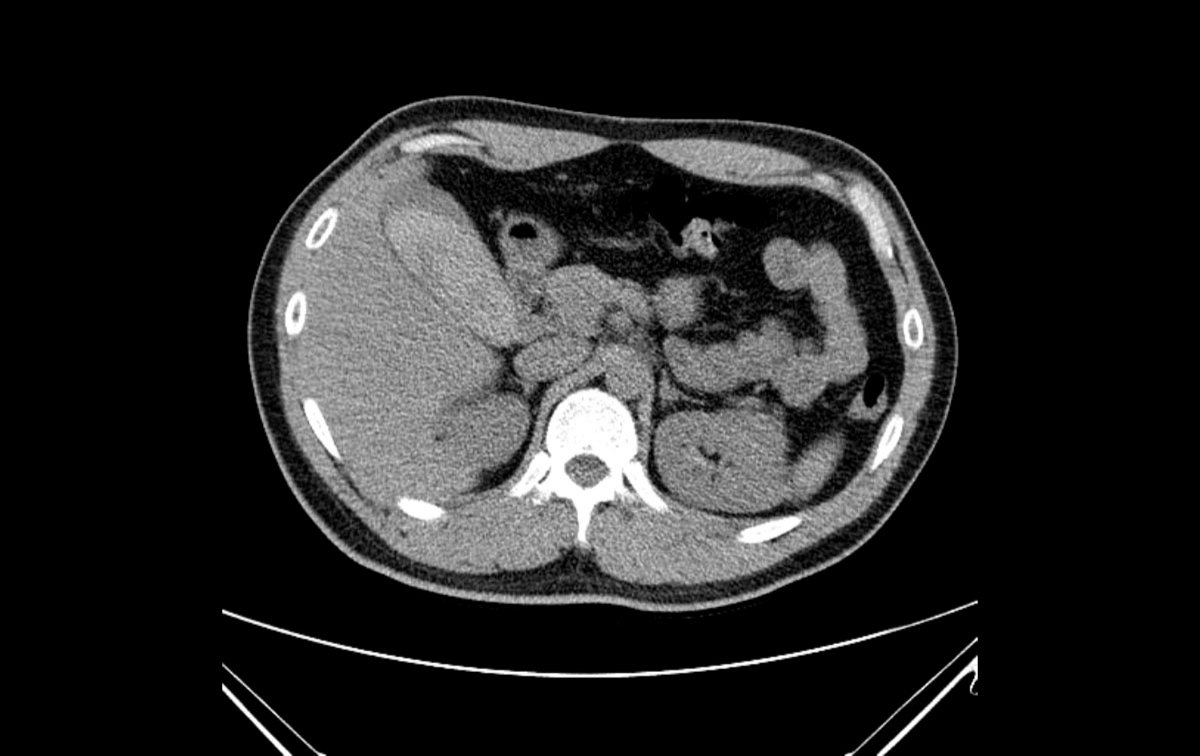
Figure 2

**Figure 3:**
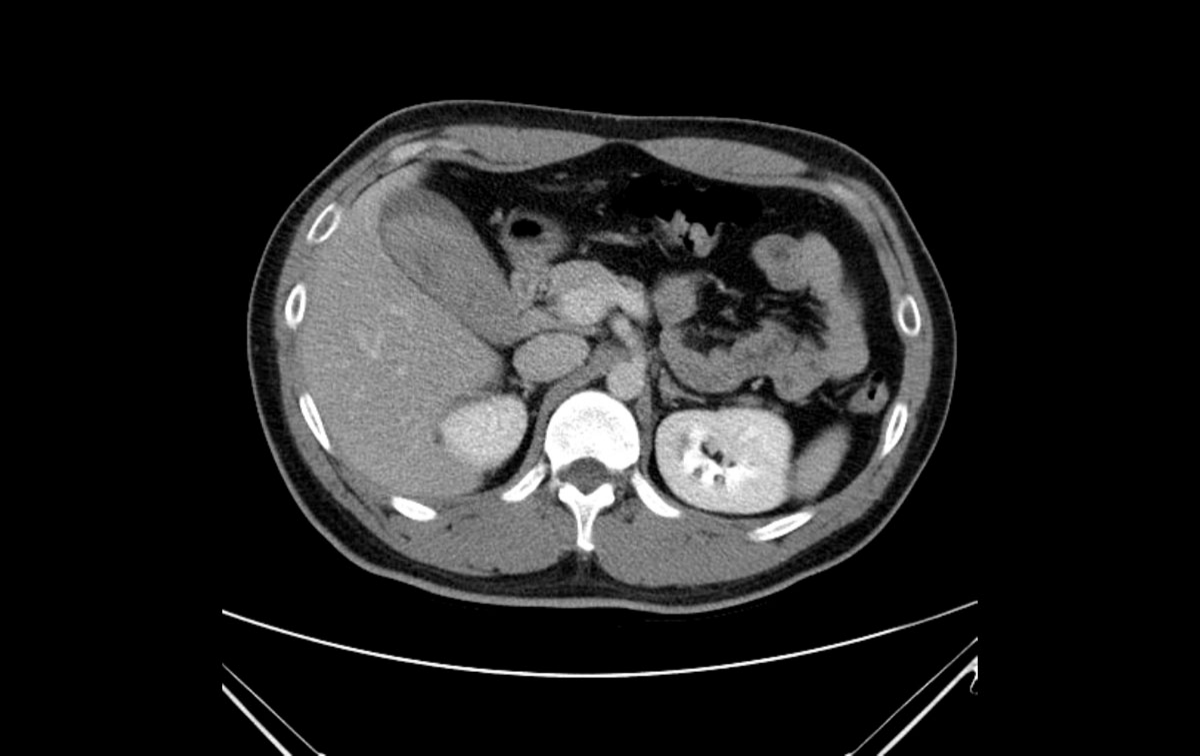
Figure 3

**Figure 4:**
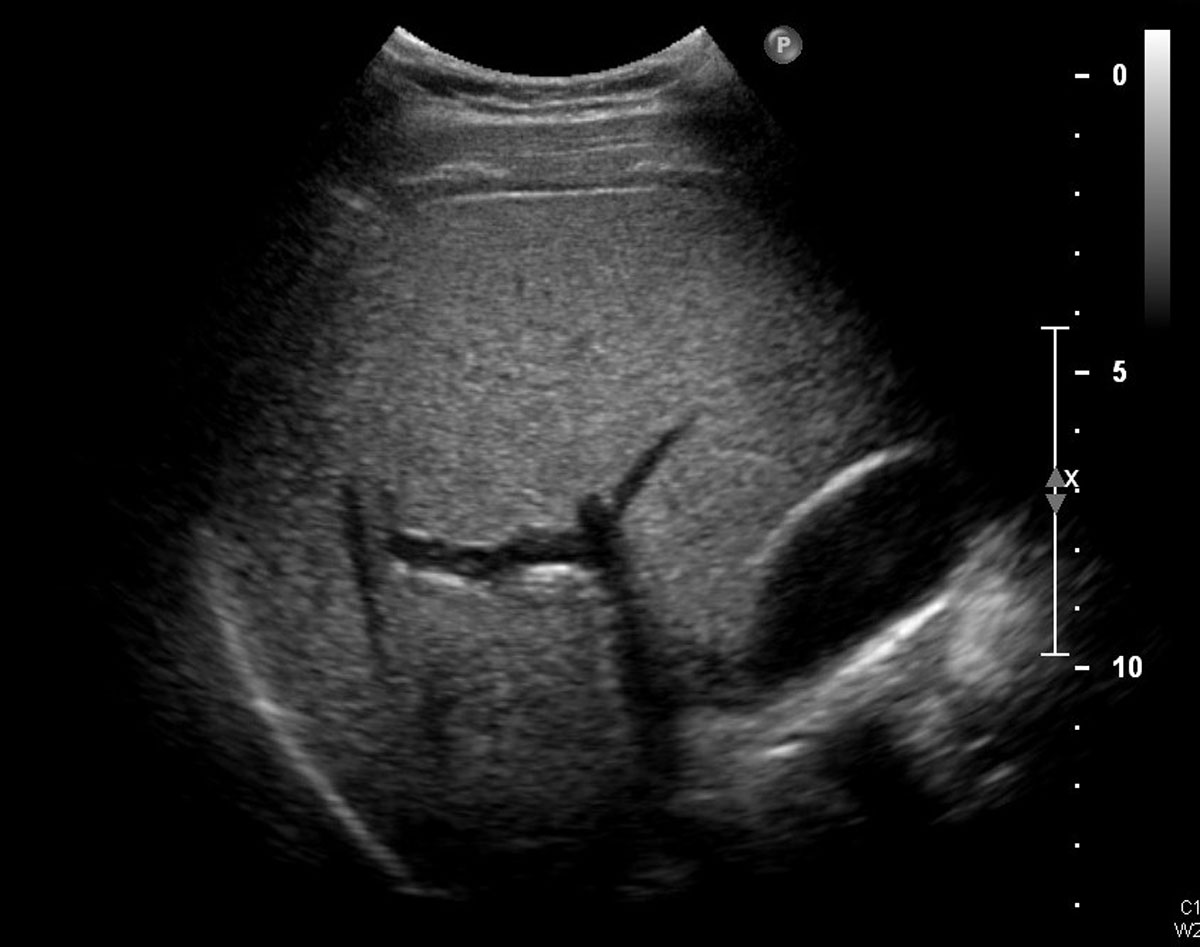
Figure 4

A 29-year-old previous healthy man was a motorcycle driver, and he bumped into another motorcycle one day ago. He visited our emergency department due to progressive right upper abdominal pain. He denied fever or bloody stool. Initial vital signs showed a body temperature of 35.4°C, a blood pressure of 147/86 mmHg, and a pulse rate of 56/min. Physical examination revealed right upper abdominal tenderness on palpation with positive of Murphy’s sign. No obvious ecchymosis was noted during inspection. The remainder of the physical examination was unremarkable. Laboratory findings revealed an elevated hepatic enzyme level with a serum Glutamic Pyruvic Transaminase (GPT) titer of 68 IU/L. The point of care abdominal sonography was performed and demonstrated heterogenic hyperechoic lesion without acoustic shadow in gallbladder. Gallbladder wall is normal and no obvious pericholecystic fluid accumulation (Figure 1). Gallbladder hematoma was impressed. Abdominal computed tomography also showed hyper-dense substance in gallbladder without liver injury (Figures 2 and 3). Patient was admitted for conservative treatment, due to patient refused operation. Abdominal pain subsided, and he discharged 3 days after admission. Follow-up sonography after 1-month revealed normal appearance of gallbladder without hyperechoic lesion inside (Figure 4).

## Discussion

Cases of gallbladder injury happen mostly in penetrating abdominal trauma and rarely in blunt trauma [2, 5]. According to Chad's study, only 2% of gallbladder injuries were isolated, while in associated injuries, approximate 49% of them were severely injured with unstable hemodynamic status [4]. All of the gallbladder injuries received invasive treatment with 93% cholecystectomy and 7% percutaneous drainage [4]. Currently, gallbladder injuries include contusion, laceration, and avulsion. Among these injuries, only contusion type is not communicated to intraabdominal space. Gallbladder contusion was commonly defined as intramural hematoma. However, in our case, the diffuse high-echogenicity in the whole gallbladder demonstrated the possibly rended of muscularis layer of gallbladder; therefore, minor tear of gallbladder should also be concerned. Under such injury force, no presentation of bruise, laceration or abrasion wound is the pitfall on this case. The missing diagnosis should be aware, and surgical exploration should be intervened.

## Informed consent

The study was conducted in accordance with the ethical standards dictated by applicable law. Informed consent was obtained from each owner to enrolment in the study and to the inclusion in this article of information that could potentially lead to their identification.
